# Identification of Breast Cancer Metastasis Markers from Gene Expression Profiles Using Machine Learning Approaches

**DOI:** 10.3390/genes14091820

**Published:** 2023-09-20

**Authors:** Jinmyung Jung, Sunyong Yoo

**Affiliations:** 1 Division of Data Science, College of Information and Communication Technology, The University of Suwon, Hwaseong 18323, Republic of Korea; 2 Department of ICT Convergence System Engineering, Chonnam National University, Gwangju 61005, Republic of Korea

**Keywords:** metastasis marker, gene expression, machine learning, XGBoost, breast cancer, feature importance

## Abstract

Cancer metastasis accounts for approximately 90% of cancer deaths, and elucidating markers in metastasis is the first step in its prevention. To characterize metastasis marker genes (MGs) of breast cancer, XGBoost models that classify metastasis status were trained with gene expression profiles from TCGA. Then, a metastasis score (MS) was assigned to each gene by calculating the inner product between the feature importance and the AUC performance of the models. As a result, 54, 202, and 357 genes with the highest MS were characterized as MGs by empirical *p*-value cutoffs of 0.001, 0.005, and 0.01, respectively. The three sets of MGs were compared with those from existing metastasis marker databases, which provided significant results in most comparisons (*p*-value < 0.05). They were also significantly enriched in biological processes associated with breast cancer metastasis. The three MGs, SPPL2C, KRT23, and RGS7, showed highly significant results (*p*-value < 0.01) in the survival analysis. The MGs that could not be identified by statistical analysis (e.g., GOLM1, ELAVL1, UBP1, and AZGP1), as well as the MGs with the highest MS (e.g., ZNF676, FAM163B, LDOC2, IRF1, and STK40), were verified via the literature. Additionally, we checked how close the MGs were to each other in the protein–protein interaction networks. We expect that the characterized markers will help understand and prevent breast cancer metastasis.

## 1. Introduction

Cancer metastasis is one of the main causes of cancer mortality, accounting for approximately 90% of cancer deaths [1]. Metastatic cancers go through four steps (i.e., detachment, migration, invasion, and adhesion) and show different characteristics than primary cancers, which makes the treatment of metastasis much more challenging [2]. Drugs chosen to treat primary cancers are almost never effective against metastatic cancers [3]. Therefore, it is important to prevent primary cancer from progressing to metastatic stages.

The identification of genes that play key roles in metastasis is the beginning of its prevention. In many previous studies, differentially expressed genes (DEGs) were selected and utilized as the main strategy to identify metastasis markers. For example, Chen’s group determined 97 DEGs between primary lung cancers and lung cancer metastasized to the brain, and the involved biological functions and signaling mechanisms were identified [4]. In addition, 664 DEGs were identified by analyzing transcriptome profiling in matched breast cancer and lymph node metastatic tissues of seven patients [5]. Wei’s group elucidated 472 DEGs involved in the metastasis of renal cell carcinoma by examining the expression profiling for renal cell carcinoma patients with and without metastasis [6].

Utilizing machine learning models could also be a good alternative to DEG approaches for characterizing metastasis markers. This is because cancer metastasis, as well as cancer itself, is intricately related to multiple biological events and numerous factors, and a machine learning model is able to deal with multiple factors in a combinatorial manner [3]. However, to date, few machine learning models have been developed for characterizing metastasis markers. One of them is Metri’s work, which identified genes that discriminate metastatic from primary melanoma with AdaBoost machine learning models [7]. Wei’s group constructed support vector machines to identify marker genes associated with metastasis for cutaneous melanoma based on expression profiles [8]. In addition, Burton and colleagues compared seven kinds of machine learning models that predict metastasis outcome in breast cancer patients [9]. To predict breast cancer metastasis, Tseng’s group generated several kinds of machine learning models by using clinicopathological features such as sHER2 and CEA [10]. While exploring several related works, we noticed that one of the challenges of using a machine learning approach is the difficulty of determining a specific number of metastasis markers [3].

**Figure 1:**
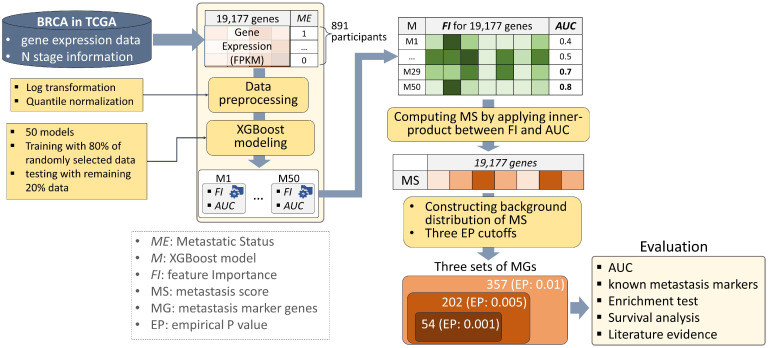
Strategy overview. We prepared gene expression and metastasis information of the 891 breast cancer (BRCA) participants obtained from TCGA. After two kinds of data preprocessing steps, XGBoost models classifying metastatic status were trained 50 times, which produced a matrix consisting of feature importance (FI) and AUC performance of the 50 models. A metastasis score (MS) was assigned to each gene by calculating the inner product between the FI and the AUC, and their significance was determined by empirical *p*-value (EP) with a background distribution of MS. Three sets of MGs were determined by three different EP cutoffs (i.e., 0.001, 0.005, and 0.01), and they were evaluated five ways, including measuring AUCs, comparing them with known metastasis markers, performing an enrichment test on processes associated with metastasis, conducting survival analysis, and exploring evidence in the literature.

In this study, we devised an algorithm that specifies a set of significant metastasis markers based on the trained machine learning models. Specifically, a scoring function was designed that calculates the inner product between the feature importance and the AUC performance of the trained models. In this study, breast cancer was selected as the cancer to be analyzed, which is the most frequently diagnosed cancer in the world [11] and has the most samples in the TCGA database. The eXtreme Gradient Boosting (XGBoost) models were trained with expression profiles of breast cancer from TCGA (BRCA), and a metastatic score (MS) was assigned to each gene by applying the devised scoring function. Then, their significance was determined using an empirical *p*-value (EP) that was obtained by comparing it to the background distribution of the MS. As a result, three sets of MGs were characterized with three kinds of EP cutoffs, which were 0.001, 0.005, and 0.01. The results were evaluated in five ways, including (1) measuring AUCs of the models built using only the characterized MGs, (2) comparing them with known metastasis markers, (3) performing an enrichment test on processes associated with metastasis, (4) conducting a survival analysis, and (5) exploring evidence from the literature. The strategy overview is depicted in Figure 1.

## 2. Materials and Methods

### 2.1. Data Preparation

Each sample of breast cancer in the TCGA database (BRCA) was given “ajcc_pathologic_m” information, indicating metastasis to other organs [12]. However, only 22 samples from the breast cancer dataset (i.e., 2% of the total samples) were classified as having a metastasis status, which was too small to use in this study. Thus, we decided to use “ajcc_pathologic_n” information instead, which indicates whether cancer is metastatic in nearby lymph nodes. There are four kinds of N stages in “ajcc_pathologic_n” information, i.e., N0, N1, N2, and N3. N0 indicates that the cancer has not spread to nearby lymph nodes, and N1, N2, and N3 indicate that the cancer has spread to nearby lymph nodes, where higher numbers indicate a higher number of lymph nodes affected by cancer. N1 is also called the micrometastasis stage, and N2 and N3 are called the macrometastasis stages [13]. In this study, N0 is referred to as M0, i.e., nonmetastatic status, and N1, N2, and N3 are referred to as M1, i.e., metastatic status. Next, FPKM expression profiles for all BRCA samples were collected and processed by using the TCGAbiolinks R package [14], and they were integrated with the metastatic information.

The expression profiles of more than 40,000 RNAs in the TCGA database include not only coding genes but also noncoding RNAs, and too many features in machine learning not only increase computational efforts but also degrade performance due to noise and redundancy [15,16]. Thus, we decided to use 19,177 genes reported in the Cancer Cell Line Encyclopedia (CCLE) [17] as features of machine learning. As a result, we obtained expression profiles of 19,177 genes from 891 (333 M0 and 558 M1) samples.

### 2.2. Data Preprocessing

The gene expression matrix was preprocessed using the following four techniques sequentially. First, gene expression was averaged per gene by mapping the ensemble IDs to gene symbols. Second, gene expression was averaged per participant by mapping the TCGA barcodes to participants. Third, log transformation was performed on every expression value to minimize outlier effects. Fourth, quantile normalization was applied to allow for an equal expression distribution for each participant (see Figure S1 for the boxplots of the preprocessed expressions).

### 2.3. XGBoost Modeling

Out of the various machine learning models, we decided to use eXtreme Gradient Boosting (XGBoost) to predict metastatic status, which is an ensemble model that has been intensively employed and has outstanding performance in biology fields [18,19]. An ensemble model combines several base models that retain good individual performance and exhibit diversities, and the XGBoost model uses a gradient-boosting algorithm that trains the base model to reduce residuals passed on from the previous base model [20]. The XGBoost models were established with the Python XGBoost package (https://xgboost.readthedocs.io/en/stable/, accessed on 14 September 2023) with their default parameters.

When an XGBoost model is being trained, feature importance (FI) scores are generated. An FI for a certain feature presents the amount of decrease in performance when it is perturbed, which is assigned to every feature while a model is being trained. A feature with a high FI indicates that it plays an important role in discriminating the class. When an XGBoost model is tested, the area under the ROC curve (AUC) is generated, which is one of the most used performance metrics in machine learning approaches [21].

### 2.4. Characterizing Metastasis Marker Genes

**Figure 2:**
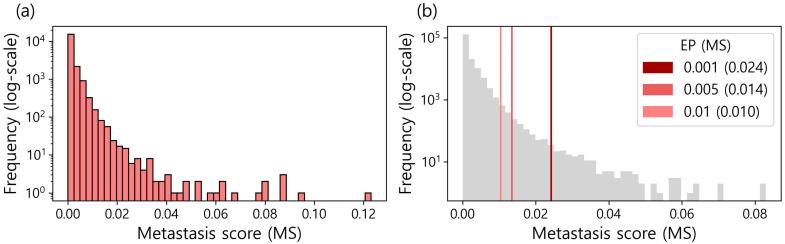
(**a**) The distribution of the metastasis score (MS) for 19,177 genes. (**b**) The background distribution of MS. It was constructed by training XGB models on the data with shuffled metastasis status. The three kinds of empirical *p*-value (EP) cutoffs (i.e., 0.001, 0.005, and 0.01) were used to characterize metastasis marker genes (MGs).

In this study, metastasis marker genes (MGs) were considered genes with the highest FI in the trained XGBoost models classifying metastasis status. This is because a high FI indicates that a gene plays an important role in discriminating metastasis status. To compute FIs, the 50 XGBoost models were constructed, each of which was trained with 80% of the randomly selected data and tested with the remaining 20% of the data. Here, we noticed that the AUCs of the 50 models varied from 0.494 to 0.692 (Figure S2). We believe that the FI of a model with a high AUC should receive a higher score than the FI of a model with a low AUC, even for the same FI. Thus, a scoring function to generate a metastasis score (MS) was designed, as shown in Algorithm 1, which calculates the inner product between the FIs and the AUCs of the 50 models. Here, the AUC is used as the weight of the FI. By applying the scoring function, we generated a set of *MSn*, where *n* = 1 to 19,177. The detailed results are presented in Table S1, and the distribution is described in Figure 2a.
**Algorithm 1.**  Inner product between the feature importance and the AUC performance of the trained modelsFor k=1 to 50:$\;\;\text{Train}\;{XGB}^{k}\;\text{with 80\% of the sampled data},\;\text{and obtain}\;{FI}_{n}^{k}\;(n=1\;\text{to}\;19,177)$$\;\;\text{Test}\;{XGB}^{k}\;\text{with remaining data}$,$\;\text{and obtain}\;{AUC}^{k}$$\text{Compute}\;{MS}_{n}:\sum_{k=1}^{50}{FI}_{n}^{k}\;\times \;{AUC}^{k}\;$ (n=1 to 19,177)*where*$\;\;{XGB}^{k}:\;k^{th}\;\text{XGB model}$$\;\;{FI}_{n}^{k}:\;\text{feature importance of}\;n^{th}\;\text{gene for}\;{XGB}^{k}$${\;\;AUC}^{k}:\;\text{AUC of}\;{XGB}^{k}$${\;\;MS}_{n}:\;\text{metastasis score of}\;n^{th}\;\text{gene}$

Significance cutoffs of the MS to determine MGs were not available. Thus, MGs were determined with an empirical *p*-value (EP) that was obtained by constructing the background distribution. To this end, Algorithm 1 was performed 10 times on the data with shuffled metastasis status, allowing the background distribution to consist of 191,770 MSs (Figure 2b). For characterizing MGs, we decided to use three kinds of EPs (i.e., 0.001, 0.005, and 0.01) as significance cutoffs, whose corresponding MSs were 0.024, 0.014, and 0.010, respectively (Table S2 and Figure 2b).

## 3. Results and Evaluations

### 3.1. Metastasis Marker Genes

**Figure 3:**
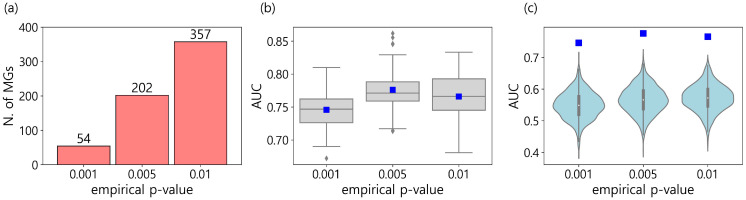
(**a**) The number of metastasis marker genes (MGs) for three empirical *p*-value (EP) cutoffs. The list of genes for each set is depicted in Table S2. (**b**) The AUC boxplots of the XGB models trained with only the characterized MGs in each of the three sets. The mean AUC is presented as a blue square (i.e., 0.746, 0.776, and 0.766 for EP cutoffs of 0.001, 0.005, and 0.01, respectively). (**c**) The AUC distributions of the XGB models trained with randomly selected genes numbering as many as the characterized MGs in each of the three sets. Blue squares are mean AUCs in Figure 3b.

As a result, three sets containing 54, 202, and 357 MGs were characterized by EP cutoffs of 0.001, 0.005, and 0.01, respectively (Figure 3a and Table S2). To evaluate the performance of the MGs, XGB models were trained using only the MGs of each set. For each set, the 50 XGB models were generated with 80% of the randomly sampled training data, and their AUCs are depicted in Figure 3b as a box plot. The mean AUCs were 0.746, 0.776, and 0.766 for each set of MGs, with EPs of 0.001, 0.005, and 0.01. We noticed that all of these AUCs were higher than 0.593, which was the mean AUC obtained using all 19,177 genes from the CCLE (Figure S2). In addition, the models using 202 genes (EP cutoff: 0.005) performed better than the models using 357 genes (EP cutoff: 0.01), which included the 202 genes with an EP cutoff of 0.005. This is consistent with the assertion that too many features in machine learning not only increase computational efforts but also degrade performance due to noise and redundancy [15,16].

Furthermore, we investigated how significant the AUCs of the MGs were when compared to those of randomly selected genes. To do this, for each of the three sets of MGs, 1000 XGBoost models were constructed with randomly selected genes, using as many as the corresponding MGs. The AUCs are depicted as boxplots in Figure 3c. We noticed that the AUC of the MGs was located at the top in all three comparisons, which indicates that the MGs were not randomly selected but had more capabilities in classifying the two kinds of metastasis statuses.

### 3.2. Comparing with Known Metastasis Markers

We evaluated the characterized MGs by comparing them with known metastasis markers. To do this, we obtained access to three metastasis marker databases, which were the Tumor Metastasis Mechanism-associated Gene Database (TMMGdb [22]), the Cancer Metastasis-related Genes database (CMGene [23]), and the Human Cancer Metastasis Database (HCMDB [24]). The TMMGdb contains 3200 genes collected with the text-mining tool BioBERT, taking into account the terms of metastatic subprocesses. The CMGene database includes 2000 genes integrated by applying a series of text-mining techniques followed by manual curation. The HCMDB contains 1900 genes obtained by collecting metastasis-related expression profiles and analyzing them. The gene lists provided by the three databases are presented in Table S3.

**Figure 4:**
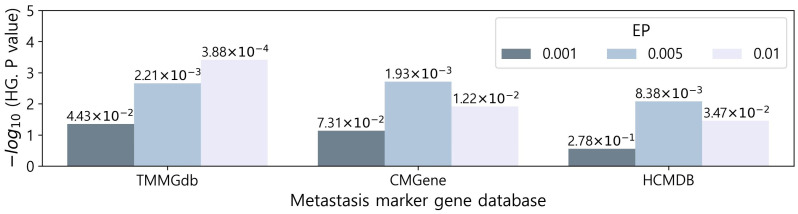
Hypergeometric test (HG) results between the characterized MGs and the genes in the three metastasis marker gene databases. EP: empirical *p*-value.

The three sets of MGs were statistically compared to the genes in each of the three databases by applying hypergeometric tests. As a result, seven of the nine comparisons produced significant results (*p*-value < 0.05), and there were three significant comparisons with a stricter *p*-value cutoff (*p*-value < 0.005) (Figure 4 and Table S4). On average, the level of significance was high in the order of EP 0.005, 0.01, and 0.001, which is the same order as the AUC result in Figure 3b.

### 3.3. Enrichment Tests on Metastasis-Related Processes

**Figure 5:**
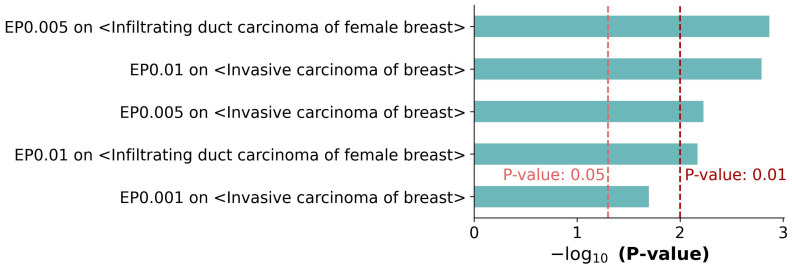
Enrichment tests on metastatic terms in the DisGeNET database. The two breast cancer metastasis-related terms in DisGeNET, “infiltrating duct carcinoma of female breast” and “invasive carcinoma of breast”, were compared to the three sets of MGs. This produced significant results in five out of six enrichment tests (*p*-value < 0.05).

DisGeNET is a discovery platform containing one of the largest publicly available collections of genes associated with human diseases, which integrates data from GWAS catalogs, animal models, and scientific literature. DisGeNET contains 1,134,942 gene–disease associations that have been identified between 21,671 genes and 30,170 diseases [25]. For each of the three sets of MGs, the MGs were evaluated by performing enrichment tests on the two breast cancer metastatic terms in DisGeNET, i.e., “infiltrating duct carcinoma of the female breast” and “invasive carcinoma of the breast”. As a result, five of the six comparisons presented with significant consequences (*p*-value < 0.05) (Figure 5 and Table S5) and four comparisons showed more significant results (*p*-value < 0.01). Similar to the previous results, the set of MGs with an EP of 0.005 showed better performance than the other two sets.

We also performed enrichment tests on the terms in KEGG and Gene Ontology. To do this, we tried to find terms related to breast cancer metastasis but were unsuccessful. Thus, we performed enrichment tests on terms associated with cancer in KEGG and Gene Ontology, and the results are depicted in Figure S3. Six out of 10 comparisons showed significant results (*p*-value < 0.05).

### 3.4. Survival Analysis

**Figure 6:**
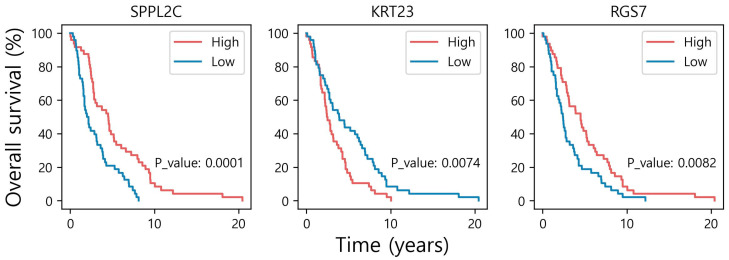
Kaplan–Meier analysis. The Kaplan–Meier plots are displayed with *p*-values of the log–rank tests for three MGs (SPPL2C, KRT23, and RGS7), which are highly significant.

We performed a survival analysis to evaluate the clinical significance of the characterized MGs. For this purpose, among the 558 patients with metastatic status, we selected the 96 patients that had survival information regarding “days_to_death” in their clinical profile. For each of the 202 MGs, Kaplan–Meier analysis and the log–rank tests were performed on the two subgroups of the 96 patients (i.e., high and low expression), which were divided based on the median expression value of the corresponding MG. Among the 202 MGs, SPPL2C, KRT23, and RGS7 showed highly significant results (*p*-value < 0.01) on the log–rank tests (Figure 6). In the literature, Ren et al. have reported that KRT23 induces migration of ovarian cancer via epithelial–mesenchymal transition [26]. In multiple previous studies, regulators of G-protein signaling (RGS) were identified as a suppressor of breast cancer migration and invasion [27,28]. The results of the three MGs with the highest MS (ZNF676, FAM163B, and LDOC1) are also displayed in Figure S4. Two of them showed significant results, with *p*-values of 0.0256 and 0.0703 for ZNF676 and FAM163B, respectively.

### 3.5. Literature Evidence

#### 3.5.1. Metastasis Marker Genes with the Highest Metastasis Score

The gene with the highest MS was ZNF676, which is closely associated with the PRMT1 gene that is involved in breast cancer metastasis [29]. The gene with the second highest MS was FAM163B, which has not yet been elucidated, but its paralog FAM163A (also known as NDSP) is associated with an increased risk for the development of cancer metastasis in bone marrow [30]. The gene with the third highest MS was LDOC2, whose function is tumor suppression that inhibits proliferation and metastasis [31]. The LDOC2 gene regulates WNT5A expression, which promotes breast cancer cell migration [32]. The gene with the fifth highest MS was IRF1, which plays a dual role in the process of the epithelial-to-mesenchymal transition (EMT). In more detail, the suppression of IRF1 in mammary epithelial cells increases the expression of mesenchymal factors; however, conversely, the inhibition of IRF1 during a TGFβ-induced EMT prevents a mesenchymal transition [33]. The gene with the eighth highest MS was STK40, whose depletion decreases cell viability and colony formation in triple-negative breast cancers (TNBCs). The knockdown of STK40 also delays tumor growth in in vivo experiments [34].

#### 3.5.2. Metastasis Marker Genes Not Identified by Statistical Analysis

The 202 MGs identified with an EP cutoff of 0.005 produced the best performance in the multiple evaluations among the three sets of MGs. Among them, we noticed that the 75 genes failed to show statistical significance when a *t*-test was performed (*p*-value > 0.1), which means that they could not be revealed by statistical analysis (refer to Table S6). We examined the literature evidence showing that they are also associated with breast cancer metastasis. For example, GOLM1 (EP: 0.0005, *t*-test *p*-value: 0.103) induces the EMT and promotes the proliferation, migration, and invasion of breast cancer cells. In addition, the overexpressing of GOLM1 markedly promotes the metastasis of breast cancer cells in vivo [35]. ELAVL1 (EP: 0.0016, *t*-test *p*-value: 0.805) was found to be modulated by MUC16, which promotes triple-negative breast cancer lung metastasis [36]. UBP1 (EP: 0.0034, *t*-test *p*-value: 0.546) consists of the CP2 transcription factor with TFCP2, which is known to be essential to the EMT process [37]. AZGP1 (EP: 0.0014, *t*-test *p*-value: 0.209) is known to reduce cell proliferation and promote invasion, and it has also been found to be a blocker of the EMT induced by the TGFbeta1-ERK2 pathway [38].

## 4. Discussion

We paid attention to the 75 MGs presented in Section 3.5.2, which were not statistically significant but were identified as MGs. One of the reasons for being characterized as MGs despite this small difference might be their biological interactions in complex molecular networks, which are combinatorial rather than individual. Thus, we checked how close the MGs were to each other in the protein–protein interaction networks. To this end, protein–protein interaction (PPI) networks were constructed from the BIOGRID database [39] by integrating protein interactions associated with affinity chromatography technology or the two-hybrid detection method, resulting in 597,215 interactions among 19,160 nodes. Then, for each of the 75 MGs, the number of adjacent MGs was calculated for the PPI network. As a result, 15 of the 75 genes were found to be involved in one or more of the adjacent MGs, such as ELAVL1 (n: 14), KRT38 (n: 5), and UBP1 (n: 3). Considering that the average number of adjacent MGs was 0.54, we can say that they had many adjacent MGs. The detailed information is depicted in Table S6. For some of them, the association with breast cancer metastasis was validated with the literature evidence presented in Section 3.5.2.

The set of MGs with an EP of 0.005, which is the middle value of the three EP cutoffs, showed a higher AUC score and better performance in all evaluations performed in this study than the other two sets. This means that, in machine learning approaches, the selection of an appropriate number of features will produce significant results. This suggests how important the cutoff decision is when selecting a specific number of features.

In this study, XGBoost, one of the ensemble models, was employed to classify metastatic status. We also trained two other kinds of ensemble models, i.e., random forest and Adaboost, by using 19,177 genes reported in CCLE, as was done for XGBoost. For each kind, 50 models were trained, and their average performances were compared. As we expected, XGBoost had the highest average AUC among the three approaches (Figure S5).

One of the limitations of this study is that it only considers one type of cancer, breast cancer. One of the obstacles to applying machine learning algorithms in the field of molecular biology is the small number of samples compared to the number of features. More samples produce more reliable results in machine learning. That is why breast cancer, which has the largest number of samples in the TCGA database, was selected as the target cancer. It would be great if the designed algorithm were applied to other cancer types, such as liver cancer and colorectal cancer, which was not possible due to insufficient data. Furthermore, the results would be empowered if the methods were applied to other datasets of breast cancer; however, it was not possible to find datasets including sufficient data such as for TCGA. Those points would be addressed in future work.

Machine learning approaches have been largely adopted in the medical community, not only in molecular biology but also in clinical fields, and they have generated many benefits. In molecular biology fields [40], machine learning models are trained to perform sequence analysis [41], protein structure prediction [42], marker discovery [43], and so on. In clinical fields [44], machine learning models are used to perform medical diagnosis [45], cancer prediction [46], medical image classification [47], and so on. In addition, data acquired through IoT and wearable sensors are being used for building machine learning models to solve various medical problems [44,48,49,50]. We hope that the methods and results of this study will contribute to further expanding the scope of machine learning applications in the medical field.

In this study, XGBoost modeling was employed to characterize a set of breast cancer metastasis markers (MGs). A metastasis score was assigned to each gene by calculating the inner product between the FIs and the AUCs of the trained models. Then, three sets of MGs were characterized by applying three empirical *p*-value (EP) cutoffs, and they were evaluated in several different ways, such as comparison with known metastasis markers, enrichment tests, survival analysis, and literature evidence. We noticed that the characterized MGs contained genes that could not be detected by *t*-tests, and we confirmed that they were also associated with breast cancer metastasis. We expect that the results of this study will be of great help in elucidating the mechanism of metastasis.

## Data Availability

Data are contained within the article or Supplementary Materials. Python implementations are available at https://github.com/jmjung83/breast_cancer_metastasis_marker (accessed on 14 September 2023).

## Supplementary Materials

The following supporting information can be downloaded at: https://www.mdpi.com/article/10.3390/genes14091820/s1, Figure S1: Boxplots of the preprocessed gene expressions for randomly selected participants (left) and genes (right); Figure S2: The box plots of the 50 AUCs obtained from the 50 trained XGBoost models; Figure S3: Enrichment tests on cancer terms in the KEGG and Gene Ontology; Figure S4: Kaplan-Meier analysis for the three MGs with highest MS (SPPL2C, KRT23, and RGS7); Figure S5: The AUC box plots of the 50 trained models with XGBoost (XGB), Random forest (RFT), and Adaboost (ADB) approach; Table S1: Metastasis scores computed with the feature importance (FI) and the AUC of the 50 XGB models; Table S2: The metastasis marker genes (MGs) determined by empirical *p*-value; Table S3: Lists of metastasis-associated genes obtained from the TMMGdb, GMGene, and HCMDB databases; Table S4: Hypergeometric tests with the three metastasis marker databases (TMMGdb, CMGene, and HCMDB); Table S5: GSEA results applied to the breast metastatic-associated terms from the DisGeNET database; Table S6: *T*-test results and the number of adjacent MGs for the 202 MGs identified with an EP cutoff of 0.005.
