# Supplementary material for: Identification of Breast Cancer Metastasis Markers from Gene Expression Profiles Using Machine Learning Approaches
Source: Genes (Basel). 2023 Sep 20;14(9):1820. doi: 10.3390/genes14091820 (PMC10530902; doi:10.3390/genes14091820)
Supplement: Supplementary file 1 [file genes-14-01820-s001.zip › supplement_Figure_S1~S5.pdf]

■ Title

Identification of Breast Cancer Metastasis Markers from Gene Expression Profiles Using Machine Learning Approaches

■ Authors

Jinmyung Jung[1,*] Sunyong Yoo[2,*]

1 Division of Data Science, College of Information and Communication Technology, The University of Suwon, Hwaseong 18323, Republic of Korea

2 Department of ICT Convergence System Engineering, Chonnam National University, Gwangju 61005, Republic of Korea

* Correspondence: jmjung@suwon.ac.kr (J.J.); syyoo@jnu.ac.kr (S.Y.)

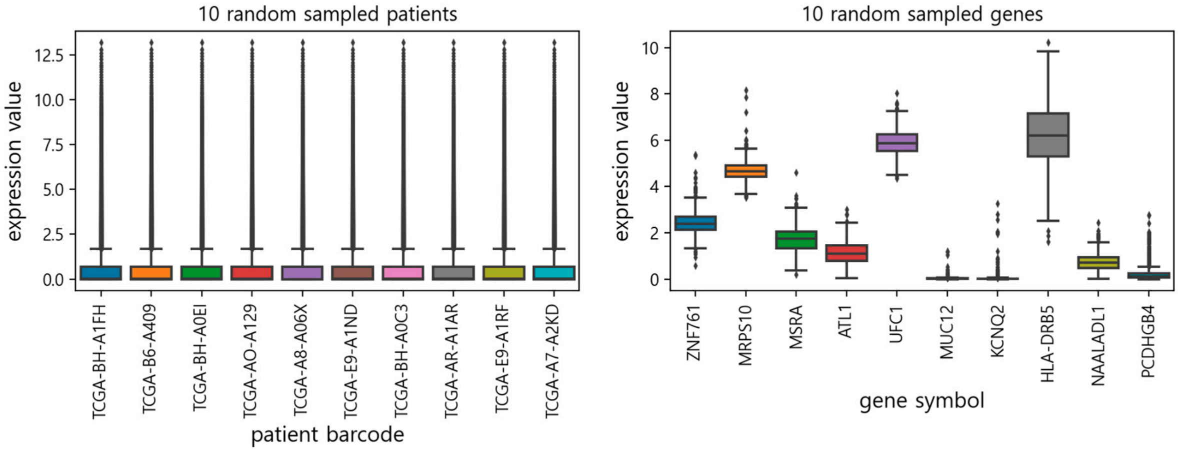

Figure S1. Boxplots of the preprocessed gene expressions for randomly selected participants (left) and genes (right)

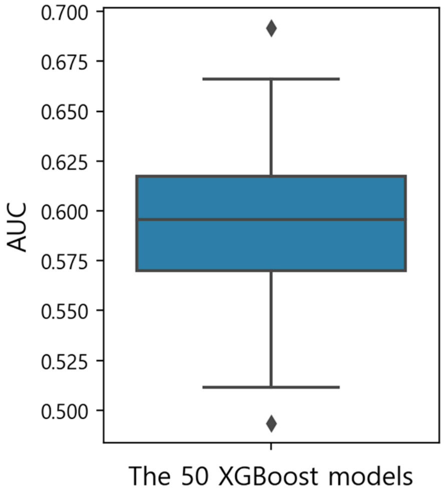

Figure S2. The box plots of the 50 AUCs obtained from the 50 trained XGBoost models.

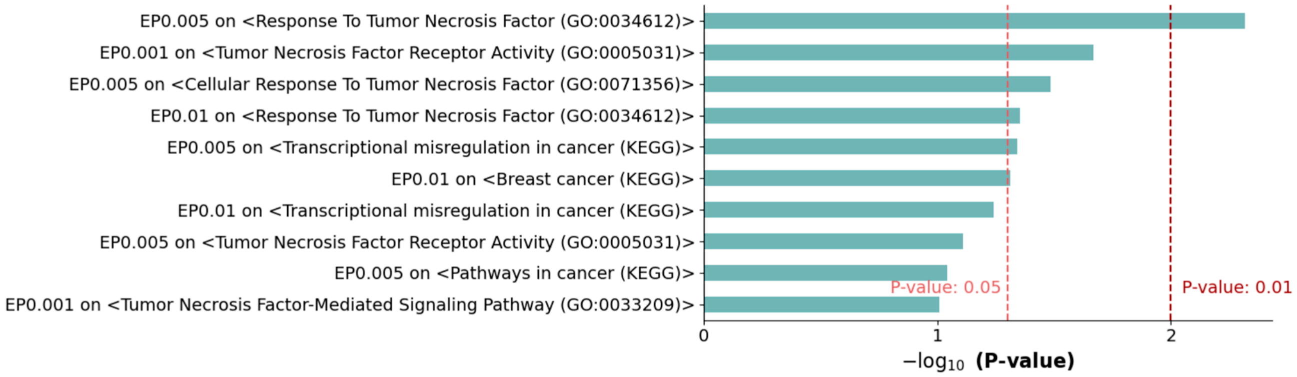

Figure S3. Enrichment tests on cancer terms in the KEGG and Gene Ontology database. This produced significant results in six out of ten enrichment tests (P-value < 0.05).

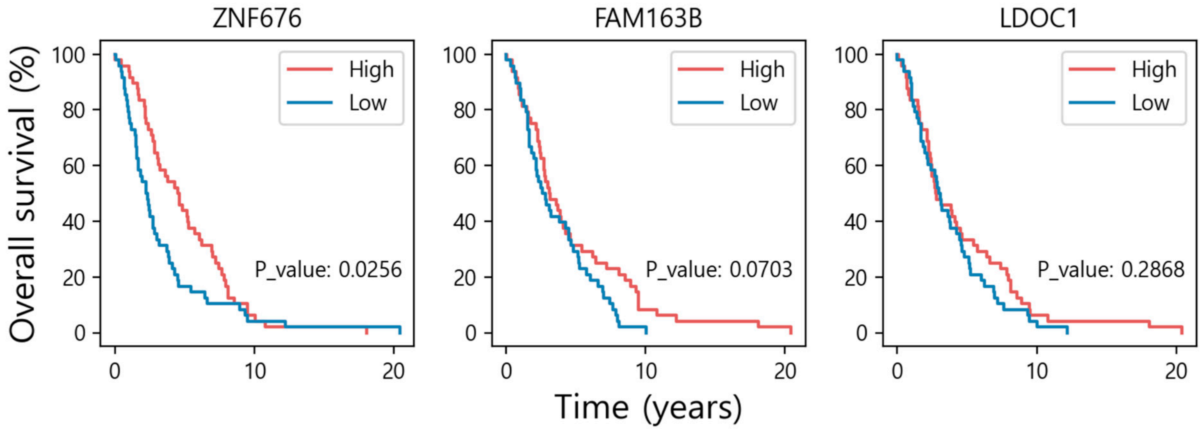

Figure S4. Kaplan-Meier analysis. The Kaplan-Meier plots were displayed with P-values of log-rank tests for the three MGs with highest MS (SPPL2C, KRT23, and RGS7).

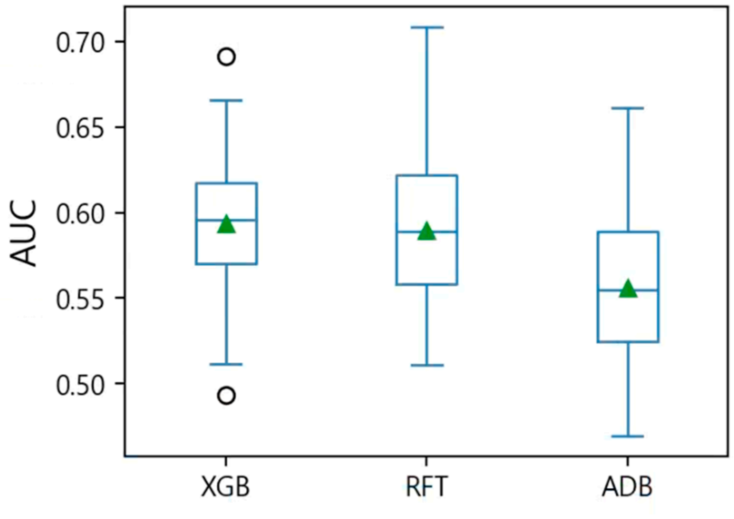

Figure S5. The AUC box plots of the 50 trained models with XGBoost (XGB), Random forest (RFT), and Adaboost (ADB) approach. The averages of the AUCs are 0.593, 0.590, and 0.556 for XGB, RFT and ADB, respectively.
